# Systematic review for the development of a core outcome set for monofocal intraocular lenses for cataract surgery

**DOI:** 10.3389/fmed.2024.1339793

**Published:** 2024-02-20

**Authors:** Rosanna Tarricone, Carla Rognoni, Anita Ciarlo, Ilaria Giabbani, Leonardo Novello, Marco Balestrieri, Giacomo Costa, Eleonora Favuzza, Rita Mencucci, Leonardo Taroni, Daniele Tognetto, Rosa Giglio

**Affiliations:** ^1^Centre for Research on Health and Social Care Management (CERGAS), SDA Bocconi School of Management, Bocconi University, Milan, Italy; ^2^Department of Social and Political Sciences, Bocconi University, Milan, Italy; ^3^Ospedale Morgagni-Pierantoni, AUSL della Romagna, Forlì, Italy; ^4^Eye Clinic, Department of Neurosciences, Psychology, Drug Research and Child Health (NEUROFARBA), University of Florence, Florence, Italy; ^5^University Eye Clinic, Department of Medicine, Surgery and Health Sciences, University of Trieste, Trieste, Italy

**Keywords:** cataract, monofocal IOLs, patients’ preferences, core outcome set, stakeholders

## Abstract

**Introduction:**

The aim of the study was to define a core outcome set (COS) to be measured following cataract surgery for the postoperative evaluation of monofocal intraocular lenses (IOLs). Compared to current COSs, the present work provides updates considering the advances in the technology due to the development of new generation monofocal IOLs, which are characterized by a safety profile comparable to standard monofocal IOLs but with an extended range of intermediate vision.

**Methods:**

Healthcare professionals (ophthalmologist surgeons) and patients were involved in the selection of outcomes to be included in the COS, starting from a list of indicators retrieved from a systematic literature search. The search considered observational studies with both a retrospective or prospective design, case studies and classic randomized controlled trials (RCTs). A mixed methodology integrating a Delphi-driven and an expert panel approach was adopted to reach an agreement among clinicians, while patients were involved in the completion of a questionnaire.

**Results:**

The final COS included 15 outcomes. Eleven outcomes, all clinical, were considered for inclusion after a joint discussion among ophthalmologists; seven outcomes were linked to visual acuity, while the remaining to contrast sensitivity, refractive errors, aberrations and adverse events. Measurement metrics, method of aggregation and measurement time point of these outcomes were specified. The most important aspects for the patients were (1) quality of life after cataract surgery, (2) the capacity to perform activities requiring good near vision (e.g., reading), (3) spectacle independence, and (4) safety of movements without fear of getting hurt or falling (intermediate vision).

**Discussion:**

In a context with limited healthcare resources, it is important to optimize their use considering also the preferences of end-users, namely patients. The proposed COS, developed involving both ophthalmologists and patients, provides an instrument for the postoperative evaluation of different technologies in the context of monofocal IOLs, which can be used not only in clinical trials but also in clinical practice to increase the body of real-world evidence.

## 1 Introduction

The struggle between the scarcity of resources and the supplier-induced demand derived from the rapid pace of innovations in healthcare can be dealt with, at least partially, by prioritizing those technologies whose benefits are relevant for all stakeholders, primarily patients. This would imply broadening the conventional measurement of clinical endpoints, often derived from randomized controlled trials (RCT), identifying and evaluating patients’ preferences. The United Kingdom’s National Institute for Health and Care Excellence (NICE) was recently forced to reevaluate its choice to employ an endovascular intervention (EVAR) for an abdominal aortic aneurysm due to the failure to consider patients’ feedback into account (1, 2). Despite the initial negative recommendation based on RCT evidence and cost modelling (1), NICE accepted EVAR as a reasonable option for disease management in line with the perspective of the scientific community and patients, who expressed firm preferences for a minimally invasive approach (2). Unfortunately, the identification and measurement processes of relevant outcomes for patients and other stakeholders is a very complex task. In this context, the core outcome set (COS) represents a consensus-derived minimum set of outcomes that should be measured and reported in research regarding a specific disease or treatment (3, 4). A COS should identify selected outcomes that are relevant to key stakeholders, like patients, clinicians and, more in general, health service users, and should drive research in the direction of meaningful and standardized evidence generation.

COS are particularly useful in research fields where there is significant variation in the outcomes measured and reported or where there is a scarcity of data available on important patient outcomes such as quality of life, long-term parameters, patients’ preferences or economic aspects of treatments. A disease that recalls these characteristics is cataract, a common condition consisting of the clouding of the crystalline lens of the eye, causing a gradual but progressive decline in vision that cannot be corrected with glasses (5). If left untreated, it can result in total blindness (5). The only effective intervention to revert visual loss caused by cataract is the surgical removal of the natural opacified lens, to be replaced with an artificial intraocular lens (IOL). Cataract surgery has been estimated to be the single most common surgical procedure performed in high-income countries worldwide (6), as well as in the European Union (7). The range of IOL models now available has expanded spanning from standard to premium technologies differing in terms of attributes, pricing, and patient outcomes (8). Monofocal IOLs allow patients to focus at only one distance, and thus require patients to wear spectacles to compensate for the lack of progressive correction at different distances. Nonetheless, they avoid the unwanted optical side effects associated with multifocality (e.g., glare, starbursts and haloes), which might cause variable patient satisfaction (9, 10). Because of their lower cost, good distance vision performance, and favorable profile in terms of incidence of photic phenomena, monofocal IOLs currently represent the standard of care, being the most implanted type of lens due to their guarantee of functionality and reliability (11). More recently, a new generation of monofocal IOLs has been introduced. These IOLs are characterized by a safety profile comparable to standard monofocal IOLs but with an extended range of intermediate vision (11).

In the cataract context, the definition of a COS is underrepresented compared to other clinical fields (3). Moreover, current core outcome sets lack considering health outcomes as derived from real-world studies (12–16) and do not indicate what measurement units should be used to assess each of the outcomes included in the set. Moreover, none of the available COS has been specifically intended for the evaluation of IOLs’ performances. Therefore, our work further advances current COSs by identifying relevant outcomes as derived from real-world evidence and direct patients’ involvement, in addition to RCTs, and indicating the metrics to be used to assess each of the outcomes of the set for the postoperative evaluation of monofocal IOLs (both standard and new generation).

## 2 Materials and methods

The development of a COS consists of four methodological steps (4). First, the scope of the COS should be clarified in terms of setting (e.g., routine care or research studies), health conditions, population and intervention addressed (17). The second step aims to check whether a new COS is needed. The third step relates to the protocol definition for the development of the COS; in this regard, the COS-STAP statement was followed, which suggests the definition of the scope of the COS, stakeholder involvement, COS development plans and consensus processes (18). The last step determines the application of the protocol to obtain a specific COS.

### 2.1 Scope of the COS

**Figure 1:**
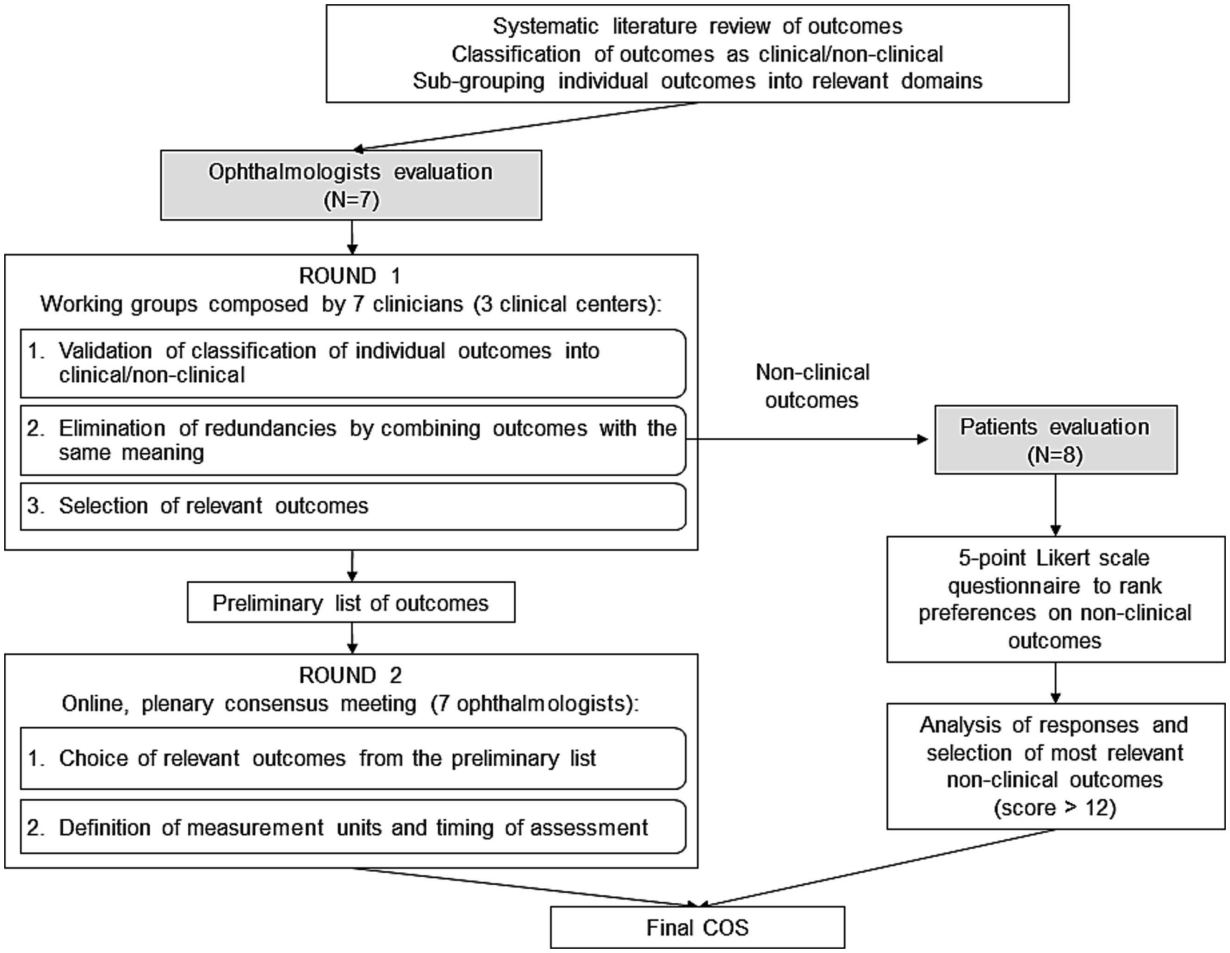
Study flowchart.

The present COS is intended to encompass key clinical and patient-reported outcomes to be measured following cataract surgery for the postoperative evaluation of monofocal IOLs. The sequential steps for the development of the COS are described in the study flow chart reported in Figure 1.

### 2.2 Need for a new COS

The proposed COS further extends previous outcome sets by (i) an extensive evaluation of outcomes (clinical and patients reported outcomes) derived through a systematic literature review involving not only RCTs but also observational (prospective or retrospective) studies; and (ii) the involvement of a sample of patients who underwent cataract surgery for the validation of patient-reported outcomes, which were not systematically evaluated in the previous studies (19, 20). Recommendations on adequate metrics and measurement criteria are also provided for each of the outcomes included in the set, offering practical guidance on how the core clinical and patient-reported outcomes should be defined and measured.

### 2.3 Protocol definition

A systematic review of all published studies on monofocal IOLs was conducted and registered in PROSPERO (CRD42022340187). The literature search was performed in the PubMed electronic database, with data cut-off June 6, 2022. Keywords were identified, resulting in the following query: (“outcome*” OR “result*” OR “indicator*” OR “effect*”) AND (“monofocal” AND ((“intraocular” AND “lens*”) OR “IOL”)) AND “cataract*.”

Observational studies with both a retrospective or prospective design, case studies and classic RCTs, were considered eligible to be included in the analysis. Instead, systematic reviews, meta-analyses, clinical guidelines, expert consensus statements, questionnaire validation studies and commentaries to previous publications were excluded. Consistently with the intended scope of the COS, the focus was restricted to studies specifically reporting post-operative outcomes related to monofocal IOL implantation in adult patients with age-related cataracts, thus excluding studies *in vitro*, on animals, on pediatric patients, focusing on the development of eye models, on the technical characteristics of IOLs devices, or exclusively on the methodology of surgical interventions.

Papers were screened by title and abstract, resulting in a first round of exclusion of records which were out of scope according to inclusion criteria. To minimize the probability of errors in paper exclusion, screening was performed independently by three researchers (AC, IG, LN), who then compared the outcomes of their selections. The remaining papers were then further screened by reading the full text, which resulted in a second round of paper selection. In case of discordance, the opinion of another researcher (CR) has been took into consideration to reach an agreement. The relevant publications were then analyzed to identify all the outcomes used to assess monofocal IOL performance in the postoperative period. The outcomes were categorized into clinical and non-clinical outcomes.

Subsequently, relevant stakeholders have been tasked to build consensus on the outcomes to be included in the COS (3). In particular, healthcare professionals (ophthalmologist surgeons) and patients were involved. Seven ophthalmologists (MB, GC, EF, RM, LT, DT, RG), 4 males and 3 females, with a mean age of 47 years (range 33–68) and average 20 years (range 7–42) of working experience (including residency), were selected among those operating in high-volume Italian public hospitals for IOL implantations (Ospedale Morgagni-Pierantoni, AUSL della Romagna, Forlì; Eye Clinic, Department of Neurosciences, Psychology, Drug Research and Child Health – NEUROFARBA, University of Florence, Florence; University Eye Clinic, Department of Medicine, Surgery and Health Sciences, University of Trieste, Trieste), while patients were reached out through Federcentri (21), the biggest Italian not-for-profit association of social and sociocultural centers for the elderly.

The full list of outcomes, grouped into main domains was submitted to the clinicians and the patients to reach consensus on the relevant outcomes to be included in the COS (17) (Figure 1).

#### 2.3.1 Ophthalmologists evaluation

A mixed methodology integrating a Delphi-driven and an expert panel approach was adopted to reach an agreement among clinicians (22). As the first step, ophthalmologists were emailed and asked to validate the grouping of outcomes, made by the researchers, in outcomes domains. As the same outcomes were defined in different publications in various ways, the next step was to eliminate outcomes redundancies.

As a third task, they were asked to select the outcomes they deemed most relevant for the assessment of monofocal IOLs by using a 2-point Likert scale (important/not important) (23).

Two or three ophthalmologists by clinical site worked together without visibility to the other ophthalmologists of the other sites. The researchers then merged all the outcomes which had been selected by at least one clinical center and prepared a preliminary set. On January 31st, 2023, an online plenary session moderated by the researchers was held to build collective consensus among the 7 clinicians on the minimum set of outcomes to be included in the final COS. Clinicians reviewed the outcomes of the preliminary set and discussed whether to include or exclude each of them in the final COS until a unanimous consensus was reached.

To make a COS actionable, it is important to define how outcomes can be measured in a standardized manner. Therefore, once the COS for monofocal IOLs was finalized, clinicians were asked to agree on how the outcomes should be measured homogeneously, specifying the domain (e.g., best corrected distance visual acuity), the specific measurement (e.g., LogMAR), the specific metric (e.g., value at a time point, change from baseline), the method of aggregation for trial data presentation (e.g., mean for continuous data) and the recommended timepoint for outcome measurement (e.g., the number of weeks post-surgery) (3, 4).

#### 2.3.2 Patients evaluation

Patients were engaged in the evaluation of non-clinical outcomes. The study has been approved by Bocconi Ethics Committee (code FA000459, approval date July 25, 2022). An anonymous questionnaire was developed and distributed to patients (Figure 1). The list of non-clinical outcomes, as retrieved from the literature and further grouped into main domains with the help of clinicians, was presented with simple words and examples so to make labelling and explanation of outcomes understandable to patients (4). Eight patients (aged >65 years, 50% males), who underwent cataract surgery with the implantation of monofocal IOL, accepted to complete the questionnaire during an in-person meeting at Federcentri in Rome on 14th April 2023 and expressed their preference on a 5-point Likert scale (23) with the following levels: not at all important, not important, quite important, important, very important.

Responses were coded with numbers expressing the relative importance of the categories (1 = quite important, 2 = important, 3 = very important, 0 otherwise) and then the sum of values was calculated across responders for each outcome. Considering 8 responders to the questionnaire, the maximum importance score for the single outcome is 24 (=8*3, i.e., “very important” level chosen by all the responders). A ranking of outcomes was obtained by ordering the calculated sums. A final list of outcomes was prepared including the ones presenting a score > 12 (higher than 50% of the maximum obtainable score) (4). The last question of the survey investigated the period (days) from cataract surgery to vision stabilization to identify the representative time for evaluating the outcomes after surgery.

## 3 Results

### 3.1 Protocol implementation

A total of 615 articles were retrieved from the PubMed database. After excluding articles that did not match the pre-defined inclusion criteria, a total of 375 studies reporting postoperative outcomes of monofocal IOLs were examined. Among these studies, 293 (78.1%) were observational – 212 with a prospective design and 81 with a retrospective design, respectively, – 71 (18.9%) were RCTs, and 11 (2.9%) were case studies. The average sample was composed of 815 individual eyes, with the median sample being 92 eyes. Further, most studies had a European affiliation, with 6% of all studies including at least one Italian research center. The outcome extraction process returned a total of 1,113 individual indicators, which were classified into 20 domains, 11 related to clinical outcomes measured by clinicians (visual acuity, accommodation, contrast sensitivity, refractive errors, aberrations, photic phenomena, positioning outcomes, general adverse events, adverse events requiring second surgery, ocular characteristic, surgeon-reported outcomes), 6 regarding patients’ reported outcomes or quality of life (patients’ satisfaction, self-reported vision, daily activities, spectacle dependence, reading performance, driving performance) and 3 focused on cost-effectiveness outcomes (cost, cost-effectiveness, effectiveness/utility). The Appendix reports all the outcomes retrieved grouped in domains. Clinical outcomes were included in every paper, while quality of life was reported in about one out of three studies and patient satisfaction in one out of five.

### 3.2 Ophthalmologists evaluation

Clinicians evaluated the full list of outcomes retrieved from the literature review. Twenty-two outcomes out of the total were selected by the three groups of ophthalmologists (Table 1, first column). Among them, ten had been included by all the groups, while twelve had been selected by only one or two groups.

**Table 1:** Core outcomes set for the evaluation of monofocal intraocular lenses from the ophthalmologists’ perspective.

Domains	List of outcomes considered at the beginning of the consensus meeting	Final outcomes chosen by ophthalmologists for the COS	Measurement unit	Metric^a^	Method of aggregation	Measurement time point (after surgery)
Aberrations	Higher order aberrations	Yes	Micrometer	Value at a time point	Mean ± SD	1 month
Adverse events	Endophthalmitis	–				
Adverse events	Posterior capsule opacification	Yes	Binary: yes/no	Value at a time point	Overall frequency (%)	6 months
Contrast sensitivity	Contrast sensitivity (Modulation Transfer Function)	Yes	Not applicable	Value at a time point	Percentage (%)	1 month
Photic phenomena	Glare	–				
Positioning outcomes	IOL decentration	–				
Positioning outcomes	IOL tilt	–				
Refractive error	Accuracy of the postoperative spherical equivalent refraction	–				
Refractive error	Astigmatism	–				
Refractive error	Deviation of spherical equivalent from intended target refraction	Yes, monocular	Diopters	Value at a time point	Mean ± SD	1 month
Second surgery	IOL explantation	–				
Surgeon reported outcome	Assessment of performance of the IOL’s fully preloaded injector system	–				
Visual acuity	Best Corrected Distance Visual Acuity (BCDVA)	Yes, monocular	LogMAR	Value at a time point	Mean ± SD	1 month
Visual acuity	Distance-Corrected Intermediate Visual Acuity (DCIVA)	Yes, monocular	LogMAR	Value at a time point	Mean ± SD	1 month
Visual acuity	Best Corrected Near Visual Acuity (BCNVA)	Yes, monocular	LogMAR	Value at a time point	Mean ± SD	1 month
Visual acuity	Defocus curve	Yes, monocular	Visual acuity (LogMAR) vs. defocus (diopters)	Value at a time point	Mean ± SD	1 month
Visual acuity	Eye dominance	–				
Visual acuity	Glare Visual Acuity	–				
Visual acuity	Halo	–				
Visual acuity	Uncorrected Distance Visual Acuity (UDVA)	Yes, monocular	LogMAR	Value at a time point	Mean ± SD	1 month
Visual acuity	Uncorrected Intermediate Visual Acuity (UIVA)	Yes, monocular	LogMAR	Value at a time point	Mean ± SD	1 month
Visual acuity	Uncorrected Near Visual Acuity (UNVA)	Yes, monocular	LogMAR	Value at a time point	Mean ± SD	1 month

a Value at a time point or change from baseline.

During the online, plenary, consensus meeting the ophthalmologists reviewed the twenty-two outcomes of the preliminary list and reached a consensus on the ones to be included in the final COS. Participants agreed to exclude eleven of the outcomes of the preliminary list since not essential. Eleven outcomes, all clinical, were then considered for inclusion in the final COS after a joint discussion. Among them, seven are linked to visual acuity, while the remaining to contrast sensitivity, refractive error, aberrations and adverse events. Concerning one of the visual acuity outcomes, clinicians deemed necessary a differentiation based on the type of IOL assessed and suggested measuring Monocular Best Corrected Intermediate Visual Acuity (BCIVA) for standard monofocal IOLs and Monocular Best Distance-Corrected Intermediate Visual Acuity (BDCIVA) for new generation monofocal IOLs with an extended range of vision. Clinicians also agreed to further specify “contrast sensitivity” as “modulation transfer function”. Finally, 11 outcomes were identified for the COS (Table 1).

Ophthalmologists were also asked to specify the measurement metrics, method of aggregation and measurement time point of the outcomes. Clinicians agreed that all measurements should be taken monocularly. They suggested measuring all outcomes one month after surgery, while the presence of posterior capsule opacification should be measured 6 months after surgery.

### 3.3 Patients evaluation

The questionnaire administered to the patients reported 49 outcomes as derived from the literature and further aggregated into main domains with the support of clinicians (see Supplementary Table S1).

In general, patients considered very important the aspects related to (1) quality of life after cataract surgery, (2) the capacity to perform activities requiring good near vision (e.g., reading), (3) spectacle independence, and (4) safety of movements without fear of getting hurt or falling (intermediate vision). Aspects related to costs were ranked as intermediate, while not important at all were the aspects related to the speed/distance of reading and pain after surgery. The full list of ordered outcomes is reported in Supplementary Table S1.

The mean time from cataract surgery to vision stabilization was 34 days (range 4–60).

## 4 Discussion

Developing and identifying technologies whose benefits are relevant to the healthcare ecosystem is crucial when prioritization is necessary to keep the healthcare systems financially sustainable. This is the principle of the value-based healthcare paradigm. Investments and market access authorization for medical technologies have often been based on clinical endpoints not necessarily relevant for end-users such as clinicians, patients and providers (24, 25). Determining what is truly crucial and for whom is therefore fundamental to guide developers’ focus on investing in technologies that are likely to deliver added value to stakeholders and decision-makers (e.g., regulatory and Health Technology Agencies bodies). This will help developers to identify innovations that can positively impact patients’ lives and the system as a whole. The use of COS has been developed to define the most relevant outcomes for all stakeholders. Although many institutions and organizations are supporting the development of relevant metrics, comprehensive and standardized identification of outcome sets has been lacking (26, 27). The COMET (Core Outcome Measures in Effectiveness Trials) initiative has started exploring the setting of clinical trials to guide the development, implementation, evaluation and updating of core outcome sets (4, 17, 18, 28). At present, considering cataract surgery, two core outcome sets have been published and are available in the COMET initiative database. Mahmud and co-authors (19) have proposed a core set of pre, intra, and post-operative measures for cataract surgery outcomes, applicable to all types of IOLs. The COS was developed through experts’ consensus, also including the perspective of a patient advocate. Subsequently, Evans et al. (20) have developed a core set by extracting and summarizing the main outcomes reported in published RCTs comparing multifocal and monofocal IOLs. Moreover, since 2012 the International Consortium for Health Outcomes Measurement (ICHOM) has started to promoting and facilitating the development of global sets of patient-centred outcome measures worldwide to create better value for all stakeholders (29). An ICHOM standard set has been proposed for cataract as well, but it does not include peculiar considerations about the IOLs performance or on the economic impact of the surgery (19). The present study aimed at developing a COS for cataract patients scheduled to standard or new-generation monofocal IOLs implantation, starting from a comprehensive literature search of reported outcomes and involving both ophthalmologists and patients. In line with current trends in health technology evaluation, which aim to compare treatment options based on patients’ preferences, patients’ input is essential for producing a COS that is as comprehensive as possible (30, 31). The literature provides examples of patient-important outcomes that were mistakenly left out when the COS was created without their participation. In the context of fibromyalgia (32), seven outcomes considered important by the patients like pain, stiffness, fatigue, sleep, patient and physician global assessment of improvement and tenderness, were not included in any of the 9 clinical studies assessed through a meta-analysis which considered clinical trials on treatment. Other important symptoms identified by the patients, such as cognition, depressive and anxiety symptoms, were not explored by most of those studies. Another example refers to rheumatoid arthritis, for which patients selected 63 treatment outcomes through in-depth interviews. Many of the 63 outcomes described as important are not currently included in the commonly used professional core sets (33). Also, in our study, ophthalmologists selected, as important, 11 outcomes which were all clinical and did not include any non-clinical outcomes, 4 of which were instead selected through the evaluation performed by patients, determining a final COS composed of 15 outcomes (see Supplementary Table S2 for the complete list of outcomes proposed for the COS). The inclusion of patients’ perspectives allowed us to broaden the analysis by considering aspects that matter to the final users of the technology. Moreover, concerning vision stabilization after cataract surgery, the timing for the evaluation of the main vision parameters proposed by the ophthalmologists (30 days) was in line with the perception of the patients (mean 34 days). Our study also suggested the measurement units for such parameters.

The present COS is an extension of previous works developed on the topic of IOLs. The search strategy was designed to identify all studies evaluating monofocal IOLs, including both single-arm and comparative assessments of monofocal IOLs against any other IOL type in both RCTs and observational studies. Indeed, the other published studies on COS development either started from the appraisal of the existing literature focused on RCTs only (20) or considered data collected through existing registries (19). Moreover, the present study actively involved patients in the assessment of key outcomes. In previous studies, the consensus on the COS was reached only among the authors of the publication (without the involvement of patients) (20) or through a group composed of clinicians and only one patient advocate (19). Visual acuity, contrast sensitivity, refractive errors, adverse events, and patients-reported quality of vision and quality of life (including spectacle independence) have been confirmed relevant for clinicians and patients like in the other published COS (19, 20). Nevertheless, our analysis pointed out the importance of assessing also clinical outcomes like aberrations and posterior capsule opacification, and patients’ reported outcomes like the ability to perform activities related to near vision without glasses and safety of movements.

Finally, while the proposed COS focuses on monofocal IOLs, it represents a valuable use case for the extension of the present study methodology to the evaluation of other devices in both ophthalmology and other disease areas.

### 4.1 Study limitations

The study has also limitations that need to be recognized. Regarding the involvement of groups of clinicians and patients, in the former, participants received feedback from their own group members, while patients, having filled in a questionnaire, did not have the chance to discuss together their opinions. Moreover, the two groups were involved separately, without considering interactions between clinicians and patients. Nevertheless, spoken language and non-verbal communication of clinicians in such meetings could exclude or undermine the participation of patients. Indeed, some COS developers recommend that face-to-face consensus meetings are to be held separately for patients and professionals to allow patients’ views to be heard without contamination from other parties (34).

Second, our study focused on post-operative outcomes only, anyway, this represents the context where standardization is needed the most, as proved by the numerous and heterogeneously measured outcomes that can be retrieved in the literature (35).

Another point relates to the measurement units of non-clinical outcomes. Patients were not tasked to identify any specific metrics since there are few already developed questionnaires for the assessment of the subjective quality of vision. An example is the Quality of Vision (QoV) questionnaire that consists of a 30-item instrument on three scales providing a QoV score in terms of symptom frequency, severity, and bothersome (36). Another instrument is the Catquest- 9SF (37), a questionnaire to be administered before and after cataract surgery that assesses visual disabilities in daily life, activity level, cataract symptoms and degree of independence. The VF-14 is instead a brief questionnaire designed to measure functional impairment caused by cataract (38).

## 5 Conclusion

In a context with limited healthcare resources, it is important to optimize their use considering also the preferences of end-users, namely patients. Moreover, the use of COS fosters cross-study comparability while discouraging selective outcome reporting, allowing for more effective data synthesis and comparison of the efficacy of different technologies. This, in turn, may increase the entire evidence base available to influence clinical practice, produce clinical guidelines, and make healthcare resource allocation choices (4), with benefits reaching throughout the healthcare system. The present study aimed at developing a core set of outcomes for the postoperative evaluation of monofocal IOLs for cataract surgery. The process, which involved both ophthalmologists and patients, showed the important role of the latter that allowed including patients’ reported outcomes, specifically quality and satisfaction in near vision without glasses, quality of life, safety of movements and reading ability. The proposed COS is intended to be measured and reported not only in clinical trials but also in clinical practice to increase the body of real-world evidence (39–41). In the case of comparative assessments, it would be crucial to also include cost-effectiveness outcomes to optimize the allocation of healthcare resources.

## Data availability statement

The original contributions presented in the study are included in the article/Supplementary material, further inquiries can be directed to the corresponding author.

## Ethics statement

The study involving humans was approved by Bocconi Ethics Committee, Bocconi University. The study was conducted in accordance with the local legislation and institutional requirements. The participants provided their written informed consent to participate in this study.

## Author contributions

RT: Conceptualization, Methodology, Supervision, Writing – review & editing. CR: Formal analysis, Investigation, Methodology, Project administration, Resources, Visualization, Writing – original draft, Writing – review & editing. AC: Formal analysis, Investigation, Writing – review & editing. IG: Formal analysis, Investigation, Writing – review & editing. LN: Formal analysis, Investigation, Writing – review & editing. MB: Investigation, Writing – review & editing. GC: Investigation, Writing – review & editing. EF: Investigation, Writing – review & editing. RM: Investigation, Writing – review & editing. LT: Investigation, Writing – review & editing. DT: Investigation, Writing – review & editing. RG: Investigation, Writing – review & editing, Supervision.

## References

[1] Overview | Abdominal aortic aneurysm: diagnosis and management | Guidance | NICE. (2020). Available at: https://www.nice.org.uk/guidance/ng156 (Accessed June 8, 2023).

[2] Winterborn RJ Amin I Lyratzopoulos G Walker N Varty K Campbell WB. Preferences for endovascular (EVAR) or open surgical repair among patients with abdominal aortic aneurysms under surveillance. J Vasc Surg. (2009) 49:576–581.e3. doi: 10.1016/j.jvs.2008.09.012 19268761

[3] Saldanha IJ Le JT Solomon SD Repka MX Akpek EK Li T. Ophthalmology outcomes working groups. Choosing Core outcomes for use in clinical trials in ophthalmology: perspectives from three ophthalmology outcomes working groups. Ophthalmology. (2019) 126:6–9. doi: 10.1016/j.ophtha.2018.09.008, PMID: 30577918 PMC6474247

[4] Williamson PR Altman DG Bagley H Barnes KL Blazeby JM Brookes ST. The COMET handbook: version 1.0. Trials. (2017) 18:280. doi: 10.1186/s13063-017-1978-4, PMID: 28681707 PMC5499094

[5] Allen D Vasavada A. Cataract and surgery for cataract. BMJ. (2006) 333:128–32. doi: 10.1136/bmj.333.7559.128, PMID: 16840470 PMC1502210

[6] Fattore G Torbica A. Cost and reimbursement of cataract surgery in Europe: a cross-country comparison. Health Econ. (2008) 17:S71–82. doi: 10.1002/hec.1324, PMID: 18186033

[7] Surgical operations and procedures statistics. Available at: https://ec.europa.eu/eurostat/statistics-explained/index.php?title=Surgical_operations_and_procedures_statistics (Accessed May 24, 2023).

[8] de Luis EB Martínez-Indart L Martínez Alday N Sacristán Egüén C Cuadros SC. Differences in intermediate vision: monofocal intraocular lenses vs. monofocal extended depth of focus intraocular lenses. Arch Soc Esp Oftalmol (Engl Ed). (2020) 95:523–7. doi: 10.1016/j.oftal.2020.06.009 32660762

[9] Yangzes S Kamble N Grewal S Grewal SPS. Comparison of an aspheric monofocal intraocular lens with the new generation monofocal lens using defocus curve. Indian J Ophthalmol. (2020) 68:3025–9. doi: 10.4103/ijo.IJO_985_20, PMID: 33229691 PMC7856977

[10] Auffarth GU Gerl M Tsai L Janakiraman DP Jackson B Alarcon A. Clinical evaluation of a new monofocal IOL with enhanced intermediate function in patients with cataract. J Cataract Refract Surg. (2021) 47:184–91. doi: 10.1097/j.jcrs.0000000000000399, PMID: 32932369

[11] Mencucci R Cennamo M Venturi D Vignapiano R Favuzza E. Visual outcome, optical quality, and patient satisfaction with a new monofocal IOL, enhanced for intermediate vision: preliminary results. J Cataract Refract Surg. (2020) 46:378–87. doi: 10.1097/j.jcrs.0000000000000061, PMID: 32050218

[12] Rush SW Omoruyi F Rush RB. Patient attitudes and desirability regarding immediate sequential bilateral cataract surgery. Clin Ophthalmol. (2022) 16:1375–81. doi: 10.2147/OPTH.S363327 35520108 PMC9064052

[13] Leung V Vanek J Braga-Mele R Punch D Jin Y-P. Role of patient choice in influencing wait time for cataract surgery. Can J Ophthalmol. (2013) 48:240–5. doi: 10.1016/j.jcjo.2013.03.008, PMID: 23931460

[14] Brennan PF Strombom I. Improving health care by understanding patient preferences. J Am Med Inform Assoc. (1998) 5:257–62. doi: 10.1136/jamia.1998.0050257, PMID: 9609495 PMC61299

[15] Swift JK Mullins RH Penix EA Roth KL Trusty WT. The importance of listening to patient preferences when making mental health care decisions. World Psychiatry. (2021) 20:316–7. doi: 10.1002/wps.20912, PMID: 34505382 PMC8429341

[16] Gärtner FR Portielje JE Langendam M Hairwassers D Agoritsas T Gijsen B. Role of patient preferences in clinical practice guidelines: a multiple methods study using guidelines from oncology as a case. BMJ Open. (2019) 9:e032483. doi: 10.1136/bmjopen-2019-032483, PMID: 31811009 PMC6924854

[17] Kirkham JJ Davis K Altman DG Blazeby JM Clarke M Tunis S. Core outcome set-STAndards for development: the COS-STAD recommendations. PLoS Med. (2017) 14:e1002447. doi: 10.1371/journal.pmed.1002447, PMID: 29145404 PMC5689835

[18] Kirkham JJ Gorst S Altman DG Blazeby JM Clarke M Tunis S. Core outcome set-STAndardised protocol items: the COS-STAP statement. Trials. (2019) 20:116. doi: 10.1186/s13063-019-3230-x, PMID: 30744706 PMC6371434

[19] Mahmud I Kelley T Stowell C Haripriya A Boman A Kossler I. A proposed minimum standard set of outcome measures for cataract surgery. JAMA Ophthalmol. (2015) 133:1247–52. doi: 10.1001/jamaophthalmol.2015.2810, PMID: 26291752

[20] Evans JR de Silva SR Ziaei M Kirthi V Leyland MD. Outcomes in randomised controlled trials of multifocal lenses in cataract surgery: the case for development of a core outcome set. Br J Ophthalmol. (2020) 104:1345–9. doi: 10.1136/bjophthalmol-2019-315410, PMID: 31959592

[21] FederCentri APS – L’Associazione dei Centri Sociali Anziani. Available at: https://federcentriaps.it/ (Accessed May 6, 2023).

[22] Rowe G Wright G. Expert opinions in forecasting: the role of the Delphi technique In: Armstrong JS, editor. *Principles of forecasting: a handbook for researchers and practitioners*. International Series in Operations Research & Management Science. Boston, MA: Springer US (2001). 125–44.

[23] Williamson PR Altman DG Blazeby JM Clarke M Devane D Gargon E. Developing core outcome sets for clinical trials: issues to consider. Trials. (2012) 13:132. doi: 10.1186/1745-6215-13-132, PMID: 22867278 PMC3472231

[24] Wale JL Chandler D Collyar D Hamerlijnck D Saldana R Pemberton-Whitely Z. Can we afford to exclude patients throughout health technology assessment? Front Med Technol. (2022) 3:796344. doi: 10.3389/fmedt.2021.796344, PMID: 35146487 PMC8821945

[25] Abelson J Wagner F DeJean D Boesveld S Gauvin F-P Bean S. Public and patient involvement in health technology assessment: a framework for action. Int J Technol Assess Health Care. (2016) 32:256–64. doi: 10.1017/S0266462316000362, PMID: 27670693

[26] Zijlmans BL van Zijderveld R Manzulli M Garay-Aramburu G Czapski P Eter N. Global multi-site, prospective analysis of cataract surgery outcomes following ICHOM standards: the European CAT-Community. Graefes Arch Clin Exp Ophthalmol. (2021) 259:1897–905. doi: 10.1007/s00417-021-05181-5, PMID: 33855602

[27] Tognetto D Giglio R De Giacinto C Dell’Aquila C Pian G Scardellato C. Cataract standard set for outcome measures: an Italian tertiary referral Centre experience. Eur J Ophthalmol. (2021) 32:11206721211018370–910. doi: 10.1177/11206721211018370, PMID: 34053333

[28] Kirkham JJ Gorst S Altman DG Blazeby JM Clarke M Devane D. Core outcome set–STAndards for reporting: the COS-STAR statement. PLoS Med. (2016) 13:e1002148. doi: 10.1371/journal.pmed.1002148, PMID: 27755541 PMC5068732

[29] Home. *ICHOM*. Available at: https://www.ichom.org/ (Accessed May 6, 2023).

[30] Drummond M Torbica A Tarricone R. Should health technology assessment be more patient centric? If so, how? Eur J Health Econ. (2020) 21:1117–20. doi: 10.1007/s10198-020-01182-z, PMID: 32301000

[31] Mainz J Kristensen S Roe D. The power of the patient’s voice in the modern health care system. Int J Qual Health Care. (2022) 34:ii1–2. doi: 10.1093/intqhc/mzac001, PMID: 35357440

[32] Arnold LM Crofford LJ Mease PJ Burgess SM Palmer SC Abetz L. Patient perspectives on the impact of fibromyalgia. Patient Educ Couns. (2008) 73:114–20. doi: 10.1016/j.pec.2008.06.005, PMID: 18640807 PMC2564867

[33] Sanderson T Morris M Calnan M Richards P Hewlett S. What outcomes from pharmacological treatments are important to people with rheumatoid arthritis? Creating the basis of a patient core set. Arthritis Care Res (Hoboken). (2010) 62:640–6. doi: 10.1002/acr.20034, PMID: 20461785 PMC2887082

[34] Potter S Holcombe C Ward JA Blazeby JM BRAVO Steering Group. Development of a core outcome set for research and audit studies in reconstructive breast surgery. Br J Surg. (2015) 102:1360–71. doi: 10.1002/bjs.9883, PMID: 26179938 PMC5034747

[35] Qin VL Conti FF Singh RP. Measuring outcomes in cataract surgery. Curr Opin Ophthalmol. (2018) 29:100–4. doi: 10.1097/ICU.0000000000000434 28937505

[36] McAlinden C Pesudovs K Moore JE. The development of an instrument to measure quality of vision: the quality of vision (QoV) questionnaire. Invest Ophthalmol Vis Sci. (2010) 51:5537–45. doi: 10.1167/iovs.10-5341, PMID: 20505205

[37] Lundström M Roos P Jensen S Fregell G. Catquest questionnaire for use in cataract surgery care: description, validity, and reliability. J Cataract Refract Surg. (1997) 23:1226–36. doi: 10.1016/s0886-3350(97)80321-5, PMID: 9368170

[38] Steinberg EP Tielsch JM Schein OD Javitt JC Sharkey P Cassard SD. The VF-14. An index of functional impairment in patients with cataract. Arch Ophthalmol. (1994) 112:630–8. doi: 10.1001/archopht.1994.01090170074026 8185520

[39] Tarricone R Boscolo PR Armeni P. What type of clinical evidence is needed to assess medical devices? Eur Respir Rev. (2016) 25:259–65. doi: 10.1183/16000617.0016-2016, PMID: 27581825 PMC9487219

[40] Tarricone R Callea G Ogorevc M Prevolnik RV. Improving the methods for the economic evaluation of medical devices. Health Econ. (2017) 26:70–92. doi: 10.1002/hec.3471 28139085

[41] Berger ML Sox H Willke RJ Brixner DL Eichler H-G Goettsch W. Good practices for real-world data studies of treatment and/or comparative effectiveness: recommendations from the joint ISPOR-ISPE special task force on real-world evidence in health care decision making. Value Health. (2017) 20:1003–8. doi: 10.1016/j.jval.2017.08.3019 28964430

