# Supplementary material for: Systematic review for the development of a core outcome set for monofocal intraocular lenses for cataract surgery
Source: Front Med (Lausanne). 2024 Feb 20;11:1339793. doi: 10.3389/fmed.2024.1339793 (PMC10912568; doi:10.3389/fmed.2024.1339793)
Supplement: Supplementary file 1 [file Table_1.docx]

Supplementary Table S1 – Non-clinical outcomes evaluated by the patients (referring to the post-operative period). In bold the outcomes valued very important (score >12).

| **Non-clinical outcomes** | **Score** |
| --- | --- |
| **Overall quality of uncorrected near vision (e.g., image quality, focus)** | **17** |
| **Impact of cataract intervention on quality of life** | **17** |
| **Satisfaction with uncorrected near vision** | **16** |
| **Confidence in moving without fear of injuries or falls** | **14** |
| **Ability in performing near tasks without glasses** | **13** |
| **Reading ability** | **13** |
| Overall quality of uncorrected far vision (e.g., image quality, focus) | 12 |
| Quality of color perception | 12 |
| Driving ability (e.g., hazard detection, parking) or need to give up driving because of vision problems | 12 |
| Overall quality of uncorrected vision (e.g., image quality, focus) during the day | 11 |
| Ability to change focus from one distance to another (e.g., from far to near) without glasses | 11 |
| Spectacle dependence during the day | 10 |
| Spectacle dependence at night/evening | 10 |
| Overall quality of uncorrected intermediate vision (e.g., image quality, focus) | 9 |
| Visual disturbances (e.g., glares, halos) | 9 |
| Ability to lead a normal social life | 9 |
| Satisfaction with corrected near vision | 8 |
| Satisfaction with corrected far vision | 8 |
| Ability to change focus from one distance to another (e.g., from far to near) with glasses | 8 |
| Mobility | 8 |
| Out-of-pocket costs (e.g., for the purchase of glasses, cleaning products, contact lenses, eye drops, specialist visits) | 8 |
| Overall quality of uncorrected vision (e.g., image quality, focus) at night/evening | 7 |
| Satisfaction with uncorrected intermediate vision | 7 |
| Ability in performing near tasks with glasses | 7 |
| Satisfaction with uncorrected far vision | 6 |
| Overall quality of corrected far vision (e.g., image quality, focus) | 5 |
| Overall quality of corrected vision (e.g., image quality, focus) at night/evening | 5 |
| Ability in performing tasks at intermediate vision without glasses | 5 |
| Overall quality of uncorrected vision (e.g., image quality, focus) under different light conditions (e.g. natural, artificial light) | 4 |
| Overall quality of corrected near vision (e.g., image quality, focus) | 4 |
| Overall quality of corrected vision (e.g., image quality, focus) during day | 4 |
| Overall quality of corrected vision (e.g., image quality, focus) under different light conditions (e.g. natural, artificial light) | 4 |
| Ability in performing far distance tasks without glasses | 4 |
| Ability in performing far distance tasks with glasses | 4 |
| Spectacle dependence for near tasks | 4 |
| Spectacle dependence for far distance tasks | 4 |
| Uncorrected reading speed | 4 |
| Overall quality of corrected intermediate vision (e.g., image quality, focus) | 3 |
| Satisfaction with corrected intermediate vision | 3 |
| Duration of glasses use (hours per day) | 3 |
| Desire to remove or change the intraocular lens (i.e., redoing surgery) | 3 |
| Ability in performing tasks at intermediate vision with glasses | 2 |
| Spectacle dependence for intermediate tasks | 2 |
| Type of glasses used (e.g., monofocal - for one distance - versus bifocal/multifocal glasses) | 2 |
| Corrected reading speed | 0 |
| Uncorrected reading distance (cm) | 0 |
| Corrected reading distance (cm) | 0 |
| Pain after surgery | 0 |
| Number of car accidents due to visual impairment | 0 |
