# Supplementary material for: Systematic review for the development of a core outcome set for monofocal intraocular lenses for cataract surgery
Source: Front Med (Lausanne). 2024 Feb 20;11:1339793. doi: 10.3389/fmed.2024.1339793 (PMC10912568; doi:10.3389/fmed.2024.1339793)
Supplement: Supplementary file 2 [file Table_2.docx]

Supplementary Table S2 – List containing the final set of outcomes (15) recommended for the COS (items 1-11 are recommended by ophthalmologists and items 12-15 by patients).

| **Outcome** | **Description** |
| --- | --- |
| 1 | Aberrations |
| 2 | Adverse events - Posterior capsule opacification |
| 3 | Contrast sensitivity (Modulation Transfer Function) |
| 4 | Refractive error - Deviation of spherical equivalent from intended target refraction |
| 5 | Visual acuity - Best Corrected Distance Visual Acuity (BCDVA) |
| 6 | Visual acuity - Distance-Corrected Intermediate Visual Acuity (DCIVA) |
| 7 | Visual acuity - Best Corrected Near Visual Acuity (BCNVA) |
| 8 | Visual acuity - Defocus curve |
| 9 | Visual acuity - Uncorrected Distance Visual Acuity (UDVA) |
| 10 | Visual acuity - Uncorrected Intermediate Visual Acuity (UIVA) |
| 11 | Visual acuity - Uncorrected Near Visual Acuity (UNVA) |
| 12 | Quality of life after cataract surgery |
| 13 | Capacity to perform activities requiring good near vision (e.g., reading) |
| 14 | Spectacle independence |
| 15 | Safety of movements without fear of getting hurt or falling (intermediate vision) |
