# Supplementary material for: Systematic review for the development of a core outcome set for monofocal intraocular lenses for cataract surgery
Source: Front Med (Lausanne). 2024 Feb 20;11:1339793. doi: 10.3389/fmed.2024.1339793 (PMC10912568; doi:10.3389/fmed.2024.1339793)
Supplement: Supplementary file 3 [file Data_Sheet_1.PDF]

APPENDIX

Detailed outcomes categories

1. Detailed list of visual outcomes

1.1. Visual acuity

- 80-point visual field test
- Add power required for corrected near visual acuity
- Amount of additional reading required for highest near visual acuity
- Arc stereoacuity
- BCCA (Best corrected contrast acuity)
- BCDVA (Best corrected distance visual acuity)
- BCIVA (Best corrected intermediate visual acuity)
- BCNVA (Best corrected near visual acuity)
- BCVA (Best corrected visual acuity)
- BDCIVA (Best distance corrected intermediate visual acuity)
- BDCNVA (Best distance corrected near visual acuity)
- BDCVA (Best distance corrected visual acuity)
- Best binocular far acuity
- Best binocular intermediate acuity
- Best distance for near vision between the degrees of myopia
- Best monocular far acuity
- BNVA (Best near visual acuity)
- BSCVA (Best spectacle corrected visual acuity)
- CDNVA (Corrected distance near visual acuity)
- CDVA (Corrected distance visual acuity)
- CDVA-UDVA defocus curve photopic
- Chromatic difference-of-focus curves
- CIVA (Corrected intermediate visual acuity)
- CNVA (Corrected near visual acuity)
- Colour vision
- Contrast visual acuity (high & low contrast)
- Contrast visual acuity (photopic & mesopic)

- Corrected visual acuity
- Corrected visual acuity at preferred reading distance
- Corrected vision different contrast/glare levels
- DCDVA (Distant corrected distant visual acuity)
- DCIVA (Distant corrected intermediate visual acuity)
- DCNVA (Distant corrected near visual acuity)
- Defocus
- Defocus curve
- Defocus equivalent
- Defocus range
- Defocus spheres
- Defocus vergence
- DOF (Depth of field)
- Depth of focus
- Depth perception
- Distance BCVA (Best corrected visual acuity)
- Distance focus
- Distance visual acuity
- Distance vision
- Distance corrected visual acuity
- Distance contrast visual acuity
- Dynamic visual acuity
- Eye dominance
- Eye dominance change
- Far-focus visual acuity
- Focal point change
- Foveal threshold (FT) of HFA
- Functional acuity contrast
- Glare visual acuity (photopic and mesopic)
- High contrast CDVA (Corrected distance visual acuity)
- High contrast UDVA (Uncorrected distance visual acuity)
- Index (illumination lev*response delay*angular visual acuity)

- Intermediate visual acuity
- Intermediate visual acuity through the distance correction
- Jaeger visual acuity
- Low contrast acuity
- Low contrast CDVA (Corrected distance visual acuity)
- Low contrast UDVA (Uncorrected distance visual acuity)
- Low contrast visual acuity (LCVA)
- Low luminance contrast acuity
- Mesopic far vision with glare
- Mean addition for best correction
- Mean binocular proximal visual acuity
- Mean deviation (MD) of Humphrey Field Analyzer
- Mean distance correction
- Mean monocular proximal visual acuity
- Mean near add to achieve best-corrected near visual acuity
- Mean near stereopsis (arcsec)
- Mean uncorrected monocular distance visual acuity
- Mean visual acuity from far to near distances
- Mean visual acuity from far to near distances with myopic correction
- Mesopic far vision
- Near add
- Near BCVA (Best corrected visual acuity)
- Distance contrast visual acuity
- Near stereoacuity
- Near UDVA (Uncorrected distance visual acuity)
- Near visual acuity
- Near vision
- Near vision with addition
- Near visual acuity through distance correction
- Negative fusional vergence
- Objective optical quality
- Ocular optical quality

- Pattern standard deviation (PSD) of HFA
- PCDVA (Preoperative corrected distance visual acuity)
- PCNVA (Preoperative corrected near visual acuity)
- Photopic contrast acuity
- Photopic far vision
- Photopic near vision
- Photopic and mesopic visual acuity
- PHVA (Pinhole visual acuity)
- Positive fusional vergence
- Postoperative and preoperative CDVA ratios
- Postoperative near acuity
- Preferred reading distance
- Raegan visual acuity
- SKILL score
- Snellen UDVA (Uncorrected distance visual acuity)
- Snellen visual acuity
- Spatial resolution threshold (distance/intermediate/near)
- Stereo acuity/stereopsis
- Stereoacuity
- Stereopsis
- Stereoscopic discrimination
- Stereovision
- Suboptimal VF results
- TCDVA (Target-corrected distance visual acuity)
- TCIVA (Target-corrected intermediate visual acuity)
- TCNVA (Target-corrected near vision acuity)
- UCDVA (Uncorrected distant visual acuity)
- UCIVA (Uncorrected intermediate visual acuity)
- UCNVA (Uncorrected near visual acuity)
- UCVA (Uncorrected visual acuity)
- UDVA (Uncorrected distance visual acuity)
- UDVA/UNVA with artificial pupil diameter

- UDVA/UNVA with induced hyperopic/myopic astigmatism
- UIDVA (Uncorrected intermediate distance visual acuity)
- UIVA (Uncorrected intermediate visual acuity)
- UDVA (Uncorrected distance visual acuity)
- UIVA (Uncorrected intermediate visual acuity)
- UNVA (Uncorrected near visual acuity)
- Uncorrected near vision with distance correction
- Uncorrected stereoacuity threshold
- Uncorrected stereopsis
- Uncorrected vision distance eye
- Uncorrected vision near eye
- Visual function (VF) plots
- Visual acuity with glare
- Visual field
- Visual field sensitivity
- Visual function
- Visual sensitivity as assessed with SAP size III and size V
- Vitreous chamber fluid
- With and without correction

1.2. Accommodation

- Accommodation amplitude
- Accommodation curve
- Accommodative amplitude
- Accommodative power
- Accommodative response
- Accommodative range
- Apparent accommodation
- Apparent accommodation amplitude
- Change in objective accommodation at near (D)
- Convergence

- Mean amplitude of accommodation
- Mean amplitude of apparent accommodation
- Mean near point in diopters
- Mean region of accommodation (D)
- Near point of accommodation (NPA)
- Near point of apparent accommodation
- Near point of convergence
- Objective accommodation
- Pseudo-accommodative amplitude
- Pseudo-accommodation
- Pseudophakic accommodation
- Range of accommodation
- Subjective accommodation amplitude
- Subjective accommodation

1.3. Contrast sensitivity

- Binocular CS (Contrast sensitivity)
- Bright/dark environment
- CS (Contrast sensitivity)
- Contrast sensitivity function (CSF)
- Contrast sensitivity threshold(s)
- Contrast threshold for recognizing letters in the presence of glare rings
- Contrast threshold under photopic conditions (FrACT)
- Contrast sensitivity (distance)
- Contrast sensitivity (near)
- Contrast sensitivity under different luminance levels
- Contrast sensitivity under glare conditions
- Contrast sensitivity under mesopic conditions
- Contrast sensitivity under mesopic conditions with glare
- Contrast sensitivity under photopic conditions
- Contrast sensitivity under photopic glare conditions

- Contrast sensitivity under scotopic conditions
- Contrast sensitivity with glare
- Contrast sensitivity in bright light
- Contrast sensitivity in dim light
- Contrast sensitivity in medium light
- Contrast sensitivity day/night
- DCS (Distance contrast sensitivity)
- DCSM (Distance contrast sensitivity mesopic)
- DCSG (Distance contrast sensitivity mesopic with glare)
- DCSP (Distance contrast sensitivity photopic)
- Depth perception
- Functional contrast sensitivity
- LMCSF (Low mesopic contrast sensitivity outcomes)
- Mesopic contrast sensitivity
- Mesopic with glare contrast sensitivity
- Mesopic without glare contrast sensitivity
- Monocular contrast sensitivity
- NCSP (Near contrast sensitivity photopic)
- Near contrast sensitivity
- Photopic contrast sensitivity
- Photopic with glare contrast sensitivity
- Photopic without glare contrast sensitivity
- Scotopic contrast sensitivity
- Static and dynamic
- Subjective contrast sensitivity

2. Detailed list of visual disturbances outcomes

2.1. Refractive errors

- AE (Angle of error)
- Absolute spherical equivalent prediction error
- Absolute angle of error
- Absolute magnitude of error (D)
- Accuracy of the postoperative spherical equivalent refraction
- Achieved SE (Spherical equivalent)
- Actual refractive power (D)
- Adjusted SE (Spherical equivalent)
- Amount of surgically induced sphere
- Angle of deviation subjective/objective
- Amount of SIA
- Angle of error
- Astigmatic change
- Astigmatic correction
- Astigmatic correction threshold
- Astigmatic error
- Astigmatic tolerance
- Astigmatism
- Astigmatism (against-the-rule)
- Astigmatism (oblique)
- Astigmatism (vertical)
- Astigmatism (with-the-rule)
- Astigmatism reduction
- Astigmatism tolerance
- ATR corneal astigmatism
- Automated cylinder
- Automated refraction
- Autorefraction

- Average astigmatic correction
- Best sphere
- BRA (Back-calculated residual astigmatism)
- Central keratometry (Kc)
- Change in SE between distance and near
- Coefficient of adjustment
- Corneal astigmatism
- Corneal astigmatism variation
- Corneal multifocality
- Corneal power
- Corneal refractive power
- Corneal status
- Corneal topography keratometry
- Correction index
- Cycloplegic spherical equivalent
- Cylinder (D)
- Cylinder correction
- Cylinder error
- Cylinder outcome
- Cylinder refraction
- Cylindrical refractive error
- Deviation of spherical equivalent SE from the intended target refraction
- Difference between predicted and achieved postoperative residual sphere
- Difference vector (DV)
- Distance from intended refraction (SE)
- Emmetropia
- Equivalent sphere (D)
- Irregular corneal astigmatism
- Keratometric astigmatism change
- Keratometric cylinder
- Magnitude of error (D)
- Manifest cylinder

- Manifest refraction
- Manifest refraction error
- Manifest refraction spherical equivalent (MRSE)
- Manifest spherical equivalent (SE)
- Manifest subjective refraction
- Mean absolute error (D)
- Mean absolute error of postoperative refraction
- Mean anisometropia (D)
- Mean automated spherical equivalent refractive error
- Mean axis misalignment (D)
- Mean change of MRSE from the target refraction
- Mean cylindrical refractive power (diopters)
- Mean cylindric correction
- Mean cylindrical refractive power (D)
- Mean difference between targeted SE and mean postoperative SE
- Mean distance refraction
- Mean error (D)
- Mean estimated SE
- Mean keratometry
- Mean near ocular deviation angle (D)
- Mean post-operative axis of the negative cylinder
- Mean postoperative subjective cylinder
- Mean residual spherical equivalent (MRSE)
- Mean SE (M)
- Mean SE refractive error (D)
- Mean sphere
- Mean spheric correction
- Mean spherical equivalent power
- Mean spherical equivalent refraction
- Mean spherical refraction error
- Mean spherical refractive power (D)
- Mean spherical refractive power (diopters)

- Mean target versus achieved refraction (D)
- Median sphere measurement
- MRSE (Mean refractive spherical equivalent)
- Near distance refractive addition
- Objective refraction
- Objective SE
- Oblique astigmatism variation
- Oblique astigmatism
- Oblique corneal astigmatism
- ORA (Ocular residual astigmatism)
- PCA (Posterior corneal astigmatism)
- PCI (Partial coherence interferometry) keratometry (mm)
- Plus cylinder
- Postoperative (mean) manifest spherical equivalent
- Postoperative absolute manifest SE
- Postoperative astigmatism (cylinder power)
- Postoperative refractive absolute prediction errors (SE)
- Postoperative refractive astigmatism
- Postoperative refractive astigmatism prediction errors
- Postoperative refractive error
- Postoperative residual refractive astigmatism
- Postoperative spherical equivalent (SE) refraction accuracy
- PRA (Postoperative residual astigmatism)
- Primary astigmatism
- Predictability diagrams
- Predicted refractive power (D)
- Prediction error (PE) of postoperative refraction
- Prediction error of postop. refraction
- Refraction
- Refraction error in spherical equivalents (SE)
- Refractive adjustment
- Refractive astigmatism

- Refractive astigmatism accuracy
- Refractive astigmatism angle of error
- Refractive astigmatism variation
- Refractive cylinder (D)
- Refractive error from target refraction
- Refractive errors: cylindrical
- Refractive errors: spherical
- Refractive outcomes
- Refractive predictability
- Refractive SE
- Refractive stability
- Refractive stabilization during neuroadaptation
- Refractive status
- Refractive surprise
- Refractive tolerance
- Relative peripheral refraction
- Residual corneal astigmatism
- Residual keratometric cylinder
- Residual refractive cylinder
- Residual refractive error
- Residual SE refraction
- SE amplitude
- SE deviation from target refraction
- SE refractive accuracy
- Secondary astigmatism
- Secondary astigmatism RMS
- Signed spherical error
- Spatial frequency
- Sphere
- Spherical and cylindrical powers
- Spherical correction
- SE (Spherical equivalent)

- Spherical equivalent error
- Spherical equivalent refraction
- Spherical equivalent refractive outcome
- Spherical error
- Spherical power
- Spherical refraction
- Subjective refractometry
- Subjective refraction
- Subjective SE
- Surgically induced corneal astigmatism
- Surgically induced keratometric astigmatism
- Target refraction
- Target refractive error
- Target spherical equivalent
- Tolerance to residual refractive error
- Topographic astigmatism axis variation
- Visual disturbance index

2.2. Aberrations

- Coma
- Coma (conea)
- Coma (horizontal)
- Coma (vertical)
- Coma aberrations
- Coma RMS
- Corneal aberration
- Corneal aberrations RMS
- Corneal higher-order aberration RMS
- Corneal HOAs
- Corneal spherical aberrations
- Corneal wave-front aberration

- Esafoil
- Esd
- Fourth order higher-order aberrations
- Fourth-order aberrations MRS
- Fouth order spherical aberration
- Higher order aberrations (HOA)
- HOA RMS: high order root mean square
- HOA Root Mean Square
- HOAs
- Internal aberrations
- Internal spherical aberration
- Intraocular optical aberrations
- Intraocular/ocular/corneal spherical aberration
- Longitudinal chromatic aberration
- Low-order RMS
- Lower order aberrations
- Mean WF Aberration RMS
- Ocular aberrations
- Ocular aberrometry
- Ocular/corneal high order aberrations (HOA)
- Ocular/corneal spherical aberration
- Pentafoil
- Peripheral/central aberration
- Point spread function (PSF)
- Postoperative primary spherical aberrations
- Primary coma RMS
- Primary spherical aberration
- Primary spherical aberration RMS
- Quadrafoil
- Quadrifoil
- RMS (Root mean square)
- RMS coma-like

- RMS high-order wavefront aberration
- RMS higher-order
- RMS lower-order
- RMS of second order tilt
- RMS of the total coma
- RMS of the total trefoil
- RMS primary spherical
- RMS spherical aberrations
- RMS vertical C horizontal coma
- RMS vertical C oblique trefoil
- Secondary coma RMS
- Secondary spherical aberration
- Single third order coma
- Spherical aberrations
- Spherical HOA
- Spherical HOA (cornea)
- Strehl ratio
- Strehl ratio of the cornea
- Tetrafoil
- Tetrafoil RMS
- Third order trefoil
- Third-order aberrations HOA
- Total aberrations
- Total internal HOA
- Total RMS: Total root mean square
- Total root mean square RMSH
- Trefoil
- Trefoil (cornea)
- Trefoil (horizontal)
- Trefoil (vertical)
- Trefoil aberrations
- Trefoil RMS

- Visual aberration/optical phenomenon
- Wave aberration
- Wavefront aberrations
- Wavefront measurement
- Wavefronts
- Zernike coefficients-related spherical aberration C12

2.3. Photic phenomena

- BAT (Brightness acuity test) on distance acuity
- Blurred far vision
- Blurred near vision
- Blurred vision
- Colour distortion
- Colour vision deficiency
- Colour confusion index (CCI)
- Dazzle
- Degree of cyanopsia
- Diplopia
- Distance blurring
- Distorted far vision
- Distorted near vision
- Distorted vision
- Distorted vision (distance)
- Distorted vision (near)
- Disturbed colour vision
- Diurnal fluctuation
- Double images VISION
- Double vision
- Drop in visual acuity caused by glare
- Dysphotopsia
- Flare

- Flash
- Flicker
- Floaters
- Fluctuating vision
- Forward light scatter
- Ghost images
- Ghosting
- Glare
- Glare driving towards headlights
- Glare driving towards the sun
- Glare effect
- Glare on sunny days
- Glare reading on shiny paper
- Glare reading signs in stores and supermarkets
- Glare size
- Glistening
- Glistening severity grade
- Halo
- Halo radius
- Halo size
- Hazy vision
- Indistinct vision/lack of sharpness
- Instability of vision
- Level of straylight
- Light sensitivity
- Light streak
- Light-distortion index (%)
- Light-distortion irregularity (mm)
- Mean surface light scattering (CCT)
- Night driving glare
- Night dysphotopsy
- Night glare

- Night halos
- Night-time vision problems
- Optical distortion
- OSI (Objective scatter index)
- Polyopia
- Posterior capsular haze
- Potophobia
- Retinal straylight
- Rings
- Scattered light
- Shadowing
- Simultaneous vision
- Starbursts
- Streaks/stars/flare
- Unusual shadows
- Unwanted images
- Visual disturbances at night

3. Detailed list of positioning outcomes

- Agreement between pre-and postoperative alignment
- Alignment
- Anticlockwise rotation
- Axial position
- Axis
- Axis alignment (intended-final axis)
- Axis rotation
- Calibration
- Central fixation
- Centration
- Clockwise rotation
- Decentration
- Differences between estimated and clinical ELP
- Direction of rotation
- Dealignment
- Fixation
- Fixation disparity
- Fixation stability (% fixation points)
- Horizontal distance between IOL and the iris
- Horizontal tilt (degrees)
- IOL centration
- IOL decentration
- IOL dislocation
- IOL malposition
- IOL mean anterior movement
- IOL mean backward movement
- IOL mean forward movement
- IOL mean posterior movement
- IOL misalignment
- IOL movement

- IOL rotation
- IOL shift
- IOL tilt
- Lens dislocated from posterior chamber
- Lens dislocation
- Lens dislocation due to suture breakage
- Lens orientation
- Lens positioning
- Movement from the axis of implantation
- Ocular alignment/dealignment
- Off/on axis measurement
- Orientation
- Position
- Rotational stability
- Rotational angle
- Stability
- Traumatic lens dislocation
- Vertical tilt (degrees)

4. Detailed list of adverse events outcomes

4.1. General adverse events

- ACO (Anterior capsule opacification)
- Adverse event (AE) rates
- Allergic conjunctivitis
- Annular fibrosis of the anterior capsule
- Anterior capsule opacification
- Anterior capsule tear
- Anterior chamber haemorrhage
- Anterior chamber reaction
- Anterior ischemic optic neuropathy
- Bleb formation
- Blepharoplasty
- Bullous keratopathy
- Capsular contraction
- Cell deposits (macrophages)
- Cell, wound or flair complications
- Conjunctival haemorrhage
- Conjunctival injection
- Corectopia
- Corneal abrasion
- Corneal decompensation
- Corneal graft rejection and decompensation
- Corneal oedema
- Corneal operation
- Corneal scar
- Corneal stroma oedema
- Cross-linking
- Cyclitic membrane
- Cystoid macular oedema

- Damaged IOL
- Deposits on IOL
- Detachment Descemet's
- Device malfunction
- Diffractive optic dysphotopsia
- Dry age-related macular degeneration
- Dry eye
- Dysphotopsia (negative)
- Dysphotopsia (positive)
- Endophthalmitis
- Endothelial cell count (ECC)
- Endothelial cell loss (ECL)
- Endothelial decompensation
- Endothelial haze
- Entropion
- Fibrin
- Fibrin reaction
- Fibrinous inflammation
- Fibrosis of the posterior capsule
- Flare
- Glaucoma
- Glaucoma drainage device insertion
- Guttata
- Hemicentral retinal vein occlusion
- Herpes virus infection
- Herpetic keratitis
- Hypermetropic surprise
- Hypertonia
- Hyphema
- Hypopyon
- Hypopyon endophthalmitis
- Hypotonia

- Infection
- Inflammation
- Inflammatory reaction
- Interlenticular opacification
- Intraocular injections for age-related macular degeneration
- Intraocular pressure (IOP)
- Intraoperative complications
- IOL subluxation
- Iridectomy
- Iris bleeding
- Iris capture
- Iris chafing
- Iris damage
- Iris pigment dispersion
- Iritis
- Iritis UGH syndrome
- Irvine Glass Syndrome
- Lens epithelial cell (LEC) growth
- Lens opacity
- Loss of iris pigment
- Macular degeneration
- Macular fibrosis
- Macular hole
- Macular oedema
- Median percentage area of PCO
- Ocular hypertension
- Open-angle glaucoma
- Operation complication
- Pain/discomfort
- Paracentral opacity of posterior capsule
- Pars plana vitrectomy
- PCO (Posterior capsule opacification)

- Phimosis
- Pigment deposits
- Pigment dispersion
- Pigments on IOL surface
- Post-op uveitis
- Posterior capsule defects
- Posterior capsule rupture
- Posterior capsule tear
- Posterior capsulometry
- Posterior synechiae
- Posterior vitreous detachment
- Postoperative complications
- Photophobia
- Power error (incorrect lens power)
- Prolonged irritation of the anterior chamber
- Punctate keratitis
- Pupil deformation
- Pupillary block
- Pupillary capture
- Raised IOL requiring treatment
- Reduction of the anterior chamber depth
- Removed during retinal surgery
- Retinal detachment (RD)
- Retinal laser coagulation
- Retinal problems
- Retinal tear
- Retinal vein occlusion
- Rupture of the posterior lens capsule
- Scleritis
- Secondary glaucoma
- Secondary pigment glaucoma
- Shallow anterior chamber

- Shallowing of the anterior chamber
- Sight-threatening and other complications
- Steroid response with elevated IOP
- Stromal rejection
- Subluxation of the lens
- Surgical site infection
- Symptoms (burning, itching, aching, dryness)
- TASS (Toxic anterior segment syndrome)
- Time until onset
- Transient corneal oedema
- Uveitis
- Vitreous detachment
- Vitreous floaters
- Vitreous haemorrhage
- Vitreous loss
- Vitreous prolapse
- Vitreous trapped in wound
- Vitritis
- Wound complication
- Wound leak requiring revision
- Zonular damage
- Zonular dialysis

4.2. Requiring second surgery

- Capsulometry
- False add-on haptic implantation
- IOL explantation
- IOL extraction
- IOL re-positioning
- IOL repositioning rate
- IOL required exchange or explantation

- Laser-assisted subepithelial keratomileusis
- Nd:YAG capsulotomy
- Nd:YAG capsulotomy odds ratio
- Nd:YAG capsulotomy rates
- Nd:YAG laser rates
- Number of macular grid laser treatment
- Number of Nd:YAG laser posterior capsulotomy
- Probability of Nd:YAG laser treatment (hazard ratio)
- Pupil capture of IOL requiring repositioning
- Repeat IOL exchange needed
- Repeated surgical procedure
- Time from surgery to Nd:YAG capsulotomy
- Time from surgery to PCO diagnosis
- Time to macular surgery

5. Detailed list of ocular characteristics outcomes

- Absolute value of meridian misalignment
- Ambient luminance of pupil perimeter
- Angle opening distance (AOD)
- Angle recess area (ARA)
- Anterior segment exam for corneal clarity
- Anterior chamber angle
- Anterior chamber cells
- Anterior chamber depth (ACD)
- Anterior chamber depth variation
- Anterior chamber fluid
- Anterior chamber swallowing
- Anterior chamber volume
- Anterior segment abnormalities
- Anterior segment and fundus examination
- Anterior segment optical coherence tomography
- Anterior segment status
- Anterior/posterior eye segment
- Aqueous depth
- Aqueous depth distance
- Axial length
- Capsular bag diameter
- Capsular bag thickness
- Capsular bag volume
- Central corneal thickness (CCT)
- Central retinal thickness
- Change axial length
- Change in anterior chamber depth
- Changes produced on the peripapillary retinal nerve fiber layer thickness
- Ciliary apex–capsular bag plane
- Ciliary process–capsular bag distance

- Ciliary ring diameter
- Constant strabismus
- Corneal asphericity (Q-value)
- Corneal asphericity in the central 8 mm
- Corneal endothelial cell density
- Corneal endothelial cell number
- Corneal high-resolution Scheimpflug tomography
- Corneal image quality
- Corneal multifocality
- Corneal steepening
- Corneal topography
- Corneal topography keratometry
- Difference vector magnitude
- Distance/near phoria
- Epithelial healing time
- Esophoria
- Exotropia
- Eyes with meridian misalignment of 5 and 10
- Flat steep mean
- Flattening
- Foveal sensitivity threshold
- Foveal suppression
- Fundus examination
- Fundus photography
- Goldmann tonometry
- Heterophoria
- Hypermetropia
- Hyperopia (farsightedness)
- Hyperopic shift
- Hyperopic shift in spherical refractive error
- In vivo intraocular optical quality
- Intermittent strabismus

- Iris contact with IOL
- Iris defects
- Iris-ciliary process distance
- Iris–ciliary process angle
- Iris–zonula distance
- Keratic precipitates
- Lenticular opacity
- Macular optical coherence tomography
- Macular spectral-domain optical coherence tomography
- MD index
- MD zone I
- MD zone II
- MD zone III
- Mean amblyoscopic phoria
- Mean angle of exodeviation (prism diopters)
- Mean change in pupil size
- Mean mesopic pupil diameter
- Mean retinal thickness
- Mean uncorrected near exophoria angle
- Mean volume of the macular cube
- Meridian misalignment
- Microperimetry
- Modulation transfer function (MTF)
- Motor fusion
- MTF cut-off
- Myopia/hyperopia
- Myopic shift
- Near exophoria angle (prism diopters)
- Near pericentral annulus
- Objective scatter index (OSI)
- Ocular deviation
- Ophthalmoscopy

- Optic shift
- Optical biometry
- Orthophoria
- P100 amplitude
- Pachymetry
- Phacoemulsification energy
- Phoria
- Photopic high pupil diameter
- Photopic low pupil diameter
- Photopic mean pupil diameter
- Point spread function (PSF)
- Posterior capsule status
- Posterior chamber depth
- Posterior segment optical coherence tomography
- Postoperative endothelial cell count (ECC)
- Pupil size
- Pupil size (high/low photopic conditions)
- Pupil size (mesopic conditions)
- Pupil size (photopic conditions)
- Pupillometry
- Range of focus
- Rates of changes in near ocular position
- Retinal eccentricity
- Retinal image quality
- Retinal magnification
- Scheimpflug tomography
- Sclera-ciliary process angle
- Scleral spur perpendicular-sulcus distance
- Scotopic pupil diameter
- Slight myopia
- Slit lamp examination
- Slit lamp biomicroscopy

- Specular microscopy
- Straylight meter
- Straylight parameter/forward scatter
- Sulcus-to-sulcus diameter
- Suppression
- Tear film related objective scatter index (TF-OSI)
- Threshold perimetry segment
- Titmus test (100 seconds of arc)
- Titmus test (40 seconds of arc)
- Tonometry
- Topographic analysis
- Torque
- Trabecular iris angle (TIA)
- Trabecular iris space area (TISA)
- Trabecular–ciliary process distance
- Uveal biocompatibility
- View of the fundus periphery
- Visual evoked potentials pattern
- Vitreous involvement

6. Detailed list of surgeon-reported outcomes

- Assessment of performance of the IOL’s fully preloaded injector system
- Control of lens injection
- Ease of introduction
- Ease of loading
- Ease of placement in the capsular bag
- Ease of use (overall rating)
- Investigator (surgeon) satisfaction
- Manual handling

7. Detailed list of patient-reported outcomes

7.1. Satisfaction

- Bother given by photic phenomena/visual disturbances
- Clarity of vision
- Cosmetic appearance/reflection
- Dissatisfaction because of lack of visual clarity or discomfort
- Effect of bright light on uncorrected vision
- Frustrated due to visual acuity levels
- Image quality
- Improvement in comfort of vision
- Improvement in quality of vision at 6 months
- Level of disturbance of photic phenomena
- Nausea due to visual distortion
- Ocular pain
- Overall satisfaction with vision
- Pain score attributed to healing process
- Patients' impressions of their intra-ocular lenses
- Reduced range of near focus noticed
- Requested exchange due to refraction
- Satisfaction
- Satisfaction at night
- Satisfaction during day
- Satisfaction vision during day/night
- Satisfaction with correction
- Satisfaction with cost
- Satisfaction with distance vision
- Satisfaction with intermediate vision
- Satisfaction with IOL exchange
- Satisfaction with near vision
- Satisfaction with surgery

- Satisfaction with the surgical result
- Self-reported happiness in the recent past
- Self-reported overall health in the recent past
- Speed of adaptation to photic phenomena
- Tolerability of photic symptoms
- Wish to remove IOL because of photic symptoms
- Satisfaction with dist./int./near visual acuity with/without glasses
- Worry about visual acuity
- Would choose same IOL/procedure again
- Would choose the same IOL for fellow eye
- Would choose to have the procedure again
- Would recommend IOL to relatives/friends

7.2. Self-reported vision

- Clarity of vision
- Clarity of vision when driving
- Daytime outdoors vision
- Degree of trouble with vision with and without glasses
- Dependence on correction
- Difficult focus on distant objects
- Difficult focus on near objects
- Difficulties changing from the distance to the near focus
- Difficulty with colour perception
- Difficulty with distance vision
- Difficulty with near vision
- Difficulty with night vision
- Dis/int/near self-reported vision
- Distance vision, with/without glasses
- Effect of bright light on uncorrected vision
- General vision rating
- Image quality

- Indoors vision
- Near vision VF score
- Near vision, with/without glasses
- Near/far vision
- Night vision
- Night-time outdoors vision
- Peripheral vision
- Quality of night-time vision (far)
- Quality of night-time vision (intermediate)
- Quality of night-time vision (near)
- Quality of vision without glasses
- Quality of vision with glasses distant/near
- Rating of overall vision, with and without spectacles
- Rating of their vision at near range, intermediate range, distance
- Reduced range of near focus noticed
- Stated to have good quality dis/near vision
- Self-reported vision in different light conditions

8. Detailed list of quality of life outcomes

8.1. Daily activities

- Ability in activities requiring to see well up close
- Ability in applying makeup
- Ability in cell phone use
- Ability in colour distinction
- Ability in computer use
- Ability in cooking
- Ability in daily activities
- Ability in doing hobbies
- Ability in using a computer
- Accomplishing more/less
- Depth of perception (pouring coffee, etc)
- Difficulty in performing tasks (distance)
- Difficulty in performing tasks (extended intermediate distance)
- Difficulty in performing tasks (intermediate distance)
- Difficulty in performing tasks (near)
- Difficulty in performing tasks (up close)
- Difficulty in walking on uneven surfaces (e.g. cobblestones)
- Difficulty in walking up and down the stairs
- Difficulty recognizing people/objects across the street
- Difficulty shaving/applying makeup
- Difficulty walking up/down stairs
- Difficulty eating
- Difficulty in fine hand work
- Difficulty in food preparation/cooking
- Eye-hand/eye-feet coordination w/o glasses
- Limitations in going to dinner or restaurant
- Limitations in going to parties or dance
- Limitations in going to the movies or theatre

- Limitations in handwriting
- Limitations in housekeeping
- Limitations in mobility
- Limitations in near/int/distance tasks without glasses
- Limitations in playing board games or card games
- Limitations in visiting friends or relatives
- Limitations in watching television
- Limitations when doing sports
- Perceived risk of injuries and falls
- Recognizing people when they are close
- Recognizing people and objects across the street
- Role limitation
- Seeing steps or stairs
- Seeing traffic signs or store signs
- Self-reported impact of procedure on quality of life
- Self-reported difficulty in everyday life due to vision
- Self-reported difficulty reading a newspaper
- Self-reported difficulty reading a restaurant menu in dim light
- Self-reported difficulty reading ordinary print in newspapers
- Self-reported difficulty reading small print
- Self-reported difficulty reading street signs
- Self-reported difficulty reading bills
- Self-reported difficulty reading labels or prices
- Self-reported difficulty reading large print
- Social limitations
- Sports-related coordination without glasses
- VF-14 score

8.2. Spectacle dependence

- Adjusted odds ratio for spectacle independence
- Dependence always/sometimes/never

- Dependence for computer use
- Dependence for daily tasks
- Dependence for driving
- Dependence for reading signs
- Dependence for playing cards
- Dependence for reading
- Dependence for reading newspaper
- Dependence for using a computer
- Dependency during day
- Distance spectacle wear
- Duration of use of glasses per day
- Far distance spectacle wear
- Intermediate distance spectacle wear
- Near distance spectacle wear
- Near spectacle wear for small prints
- Near spectacle wear in dim light
- Night spectacle wear
- Odd ratios for likelihood of wearing distance/reading spectacles
- Performance of everyday tasks with/without glasses (near)
- Rate of spectacle use
- Spectacle independence
- Spectacle independence for distance vision
- Spectacle use out of habit
- Spectacle/contact lenses independence
- Use of bifocal glasses
- Use of distance glasses
- Use of near glasses
- Wear distance glasses for driving
- Wear distance glasses for watching TV
- Wear reading glasses always/for fine print only/never
- Wear glasses for work/hobby up close (cooking, sewing)

8.3. Reading performance

- Average reading speed
- Bright and dim light conditions
- Can read more than J2/J3
- Can read small print
- Critical print size (CPS)
- Maximum reading speed
- Mean print size patients were able to read at a speed
- Reading acuity/ability
- Reading at distance/near focus
- Reading distance (corrected/uncorrected)
- Reading performance
- Reading speed
- Reading speed based on print size
- Smallest print size that could be read with a min reading speed of 80 wpm
- Words read per minute

8.4. Driving performance

- Ability in driving
- Ability in driving at daytime
- Ability in driving at night
- Current driving habits
- Difficulty/satisfaction driving at daytime
- Difficulty/satisfaction driving at night
- Driving alone
- Driving at night
- Driving beyond immediate neighbourhood
- Driving boundaries
- Driving during rush-hour traffic
- Driving exposure/dependence

- Driving in rain or fog/difficult conditions
- Driving interstate
- Driving on high-traffic roads
- Driving on interstates
- Driving performance
- Driving performance in low contrast conditions
- Driving towards distant towns
- Driving towards neighbouring towns
- Driving when it is raining
- Driving with adverse conditions/diff. situation
- Gave up driving because of vision problems
- Gave up night driving because of vision problems
- Glasses/contact lenses dependence when driving
- Hazard avoidance behaviours while driving
- Hazard distances detection ability
- Hazard detection ability
- Immediate neighbourhood
- Parallel parking
- Percentage of correctly recognized street signs
- Self-rated quality of driving
- Self-reported crashes/citations
- Sign recognition distances
- Situational driving difficulty

9. Detailed list of economic evaluations outcomes

9.1. Cost

- Average cost per patient
- Cost difference per IOL procedure (AcrySof vs other IOLs)
- Cost for NHS
- Cost for society
- Cost of spectacles (reading/distance/multifocal)
- Cost of surgical procedure
- Cost of visits and consultation
- Costs in the years after treatment (patient)
- Costs in the years after treatment (total)
- Device cost
- Direct cost
- Direct hospital costs (monofocal/multifocal IOL)
- Direct patient costs (monofocal/multifocal IOL)
- First year incurred cost (patient)
- First year incurred costs (total)
- Healthcare costs
- Healthcare provider costs
- Incremental costs (multifocal vs. monofocal)
- Incremental device cost
- Lifetime costs (patient)
- Lifetime costs (total)
- Mean total cost of wearing spectacles
- Out-of-pocket costs
- Savings
- Societal costs
- Spectacle costs for NHS
- Spectacle costs for patients
- Spectacle costs for private insurance schemes

- Spectacle costs for society
- Total annual cost savings with the use of AcrySof over other IOLs
- Total costs per patient

9.2. Cost-effectiveness

- CER (Cost-effectiveness ratio)
- Cost per patient achieving spectacle independence
- Cost per patient achieving no night vision limitations
- Cost per patient achieving no vision limitations
- Cost per patient achieving no visual symptoms
- Cost-effectiveness probability based on ceiling ratios
- ICER (Incremental cost-effectiveness ratio)

9.3. Effectiveness/Utility

- Health-related utility
- QALYs
- Survival rate
- Survival time
- Total lifetime QALYs
- Utilities of monofocal and multifocal IOL patients
- Willingness to pay for freedom from spectacles after cataract surgery
- Willingness to pay for spectacle independence
- Willingness to pay per QALY
